# Controlled Shrinkage of Expanded Glass Particles in Metal Syntactic Foams

**DOI:** 10.3390/ma10091073

**Published:** 2017-09-13

**Authors:** Kadhim Al-Sahlani, Mehdi Taherishargh, Erich Kisi, Thomas Fiedler

**Affiliations:** School of Engineering, the University of Newcastle, Callaghan 2308, Australia; Kadhim.Al-Sahlani@uon.edu.au (K.A.-S.); mehdi.taherishargh@newcastle.edu.au (M.T.); erich.kisi@newcastle.edu.au (E.K.)

**Keywords:** metal syntactic foams, expanded glass particles, A356 aluminum alloy, particle shrinkage, infiltration, mechanical characterization

## Abstract

Metal matrix syntactic foams have been fabricated via counter-gravity infiltration of a packed bed of recycled expanded glass particles (EG) with A356 aluminum alloy. Particle shrinkage was studied and has been utilized to increase the particles’ strength and tailor the mechanical properties of the expanded glass/metal syntactic foam (EG-MSF). The crushing strength of particles could be doubled by shrinking them for 20 min at 700 °C. Owing to the low density of EG (0.20–0.26 g/cm^3^), the resulting foam exhibits a low density (1.03–1.19 g/cm^3^) that increases slightly due to particle shrinkage. Chemical and physical analyses of EG particles and the resulting foams were conducted. Furthermore, metal syntactic foam samples were tested in uni-axial compression tests. The stress-strain curves obtained exhibit three distinct regions: elastic deformation followed by a stress plateau and densification commencing at 70–80% macroscopic strain. Particle shrinkage increased the mechanical strength of the foam samples and their average plateau stress increased from 15.5 MPa to 26.7 MPa.

## 1. Introduction

Metal syntactic foams (MSF) are made by dispersing hollow or porous particles in a metallic matrix. They are usually considered as closed-cell cellular materials [1,2,3]. Owing to their excellent mechanical and physical properties (e.g., high strength, stiffness, and energy absorption capacity), they have recently attracted considerable attention as materials for structural applications [4,5,6,7]. MSF properties depend on the physical and mechanical characteristics of filler materials and the properties of the metallic matrix. In a number of studies, a variety of lightweight aggregates or hollow particles, such as cenospheres, hollow spheres [8], ceramic spheres [5], and hollow metallic spheres, have been used as filler particles. Different methods have also been used to produce MSF, such as stir casting [9], squeeze casting [10] and powder metallurgy [11]. Pressure infiltration is another widely-used process owing to its competitive cost and the high filler particle volume fraction achievable [12]. In a comprehensive review, Rohatgi, et al., demonstrated the different manufacturing methods and applications of MSFs [13].

In spite of intensive research on metallic syntactic foams, high cost remains a major challenge and limits their broad application [14]. In addition, high density and relatively high strength make them unsuitable for applications where low weight and low deformation resistance are required. In order to overcome the above-mentioned limitations, low-cost and low-density filler materials can be used for the production of metallic syntactic foams. Recently, Taherishargh et al. [15,16] manufactured a new generation of lightweight cost-efficient MSF (0.72–1.09 g/cm^3^) made by counter-gravity infiltration of low-cost expanded perlite particles (≈0.18 g/cm^3^) with molten aluminum. Similar studies have utilized lightweight porous particles such as expanded vermiculite [17], pumice [18], or expanded clay [19].

Recently, expanded glass (EG) has been used in the manufacturing of syntactic foams. Their usage helps decrease the volume of general waste and supports sustainable recycling [20,21]. It offers the properties desired for manufacturing MSFs (low-cost, low density, and good strength). EG particles are almost spherical in shape and mainly composed of closed pores which impart good acoustic insulation, thermal insulation, fire resistance, moisture resistance, and chemical inertness [22,23]. The combination of these properties makes EG a suitable candidate for applications, such as sandwich panels, lightweight concrete, mineral moulding [24], sound absorption boards [24,25], and crash box energy absorbers [26,27]. The production of EG commences with ultra-fine crushing of recycled broken glass. Fine-ground glass and binder are blended with a foaming agent and formed into granules in a centrifugal device. Then the particles are sintered and foamed (expanded) in a rotary kiln at temperatures of 750–900 °C [20,23]. 

Recently, Wright et al. [28] used EG particles with an Al-12%Si matrix to manufacture metal syntactic foams using a vacuum casting process. They produced samples with a density as low as 1.14 g/cm^3^. Therein it was observed that the mechanical properties are strongly affected by the preheat temperature of the mould containing the EG. The strength of the material decreases with increasing preheat temperature while energy-absorbing characteristics improve. According to those authors, at preheating temperatures above 400 °C clamping between the sample and the stainless steel mould occurs. The reason is a thermal expansion mismatch and subsequent sample removal damages the EG particles, resulting in decreased foam strength [28].

One noteworthy behaviour of EG particles is shrinkage during heat exposure above 600 °C. This effect is usually considered as an impediment when using EG in syntactic foams [29]. However, particle shrinkage simultaneously increases particles strength. The aim of the current study is to manufacture and characterize new MSF using EG filler particles. The first set of samples avoids significant particle shrinkage by limiting EG particle heat exposure (stable EG particles). A second set utilizes the particle shrinkage to modify the mechanical properties of foams (shrunk particles). The structural, microstructural, and mechanical properties of EG particles and the resulting syntactic foam are presented in this paper.

## 2. Results and Discussion

### 2.1. EG Particle Characteristics

The bulk $\left(\rho_{B}\right)$, particle $\left(\rho_{P}\right)$ and true $\left(\rho_{T}\right)$ densities of as-received, stable and shrunk EG particles with diameter of 2–2.8 mm are listed in Table 1. Comparing the densities of as-received and stable EG (held at 700 °C for 6 min) no significant deviation was found. However, shrunk EG (held at 700 °C for 20 min) exhibits a significantly higher particle density due to shrinkage. This is further visible in its increased bulk density.

**Figure 1:**
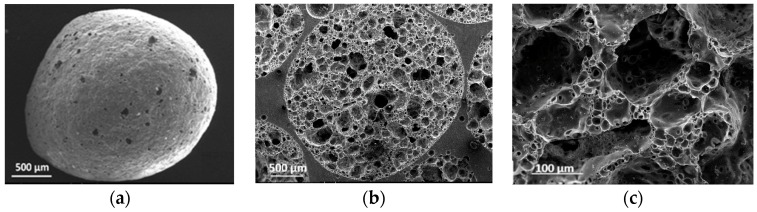
Scanning electron images of EG particle: (**a**) EG particle shell; (**b**) EG cross-section; and (**c**) pore wall.

The scanning electron microscope (SEM) images of the EG particles are shown in Figure 1a, EG particles resemble closed spheres with small shallow holes in their surface layer. In Figure 1b, a cross-section of EG particles reveals a large number of pores sized between 100 μm to 700 μm. A higher magnification in Figure 1c further indicates that the cell walls themselves contain numerous micropores 5–30 μm in size. 

**Figure 2:**
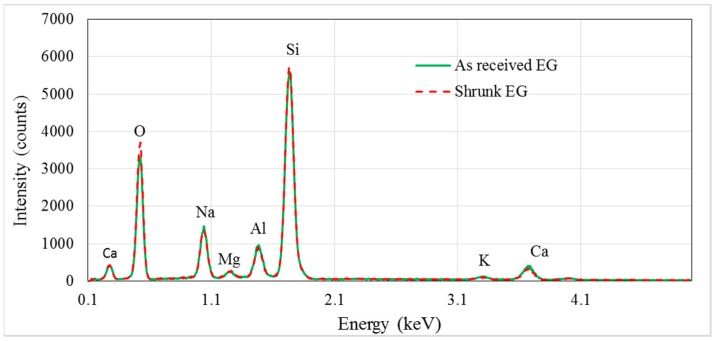
EDS spectra of raw and shrunk EG particles.

Figure 2 shows the findings of energy dispersive spectroscopy (EDS) tests on raw and shrunk EG particles. The results indicate no significant change in EG composition due to heating and shrinkage. The chemical composition of EG particles comprises 76.32 wt% SiO_2_, 7.14 wt% Na_2_O, 2.57 wt% CaO, 12.74 wt% Al_2_O_3_, 0.71 wt% K_2_O, and 0.52 wt% MgO. The densities of these oxides are 2.65 g/cm^3^, 2.27 g/cm^3^, 3.34 g/cm^3^, 3.97 g/cm^3^, 2.32 g/cm^3^, and 3.85 g/cm^3^, respectively [30]. Based on the mixing rule, the density of the solid part of the EG particles (true density) is estimated to be 2.81 g/cm^3^.

**Figure 3:**
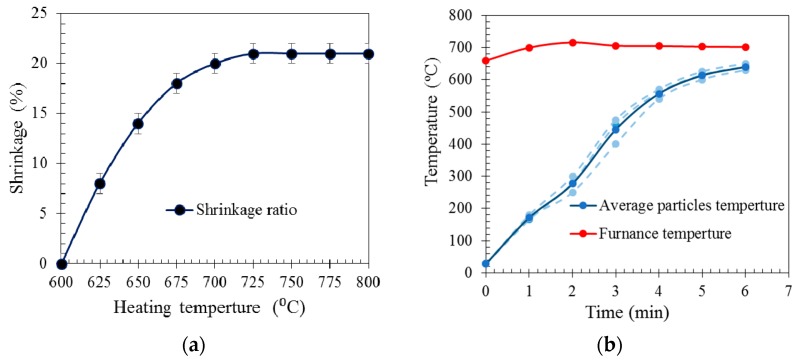
(**a**) Shrinkage ratio of EG particles for 20 min heating time at different furnace temperatures; and (**b**) the core temperature of the EG particle bed during heating.

Figure 3a illustrates the volume reduction (shrinkage) of the particles when heated for 20 min at temperatures in the range 600–800 °C. No shrinkage was observed at temperatures below 600 °C. Particles shrinkage emerged at temperatures higher than 600 °C and increased as the temperature increased to 700 °C. The shrinkage percentage remained relatively constant (21%) at temperatures between 700 °C and 800 °C. At high temperatures (>750 °C), the particles fused at their contact points. The contact points flattened and the distances between particle centroids decreased; resulting in a lower volume of packed particle bed. The volume loss of the particles bed is also attributed to shrinkage of individual particles. The particle density of the particles, which was measured by the powder technique, increased from 0.33 g/cm^3^ to 0.43 g/cm^3^ after shrinkage (see Table 1). This means that at a constant mass, the volume of individual particles reduced by 23% during heating. Assuming that 58% of the total volume is occupied by EG particles (see packing densities in Table 1), one could say that 14% of the total bulk shrinkage is attributed to volume reduction of individual particles, i.e., 23% × 58% = 14%. The rest of the shrinkage of the packed bed of particles, i.e., 21% − 14% = 7%, is due to flattening of the contact points. The data in Figure 3a clearly indicates that EG shrinkage can be avoided by limiting the furnace temperature to below 600 °C. 

The second particle shrinkage test probed the effect of heating time. To this end, EG particles were heated at 700 °C (i.e., the targeted casting temperature for A356) for gradually-increasing times. Figure 3b shows the furnace temperature and the temperature measured by a thermocouple positioned at the centre of the packed EG particles. A short initial drop of the furnace temperature is caused by opening of the furnace to insert the sample. The particle temperature increased from room temperature and converged towards the furnace temperature. The significant temperature difference between particle and furnace temperature at the initial stages of heating is due to the low thermal conductivity of EG particles. Figure 3a indicates that shrinkage only occurs above 600 °C. Hence, particle shrinkage can be prevented even at higher furnace temperatures if the exposure time (and thus particle temperature) is limited. Figure 3b shows that shrinkage of the particles in the vicinity of the thermocouple will only occur after more than 6 min of heating. 

**Figure 4:**
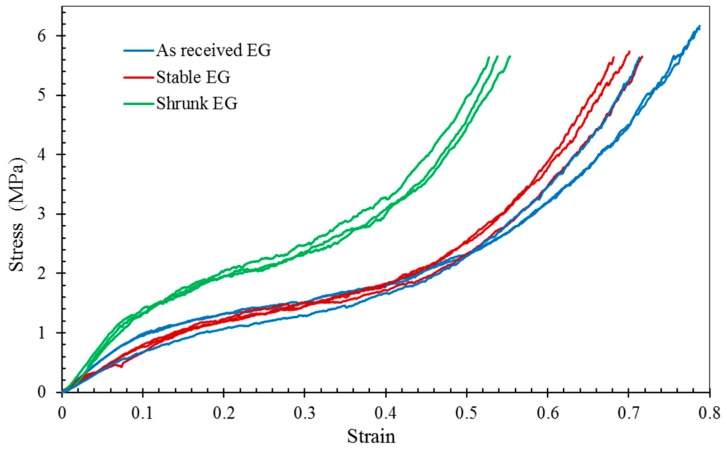
Crushing strength of as-received, stable (held for 6 min at 700 °C) and shrunk EG (held for 20 min at 700 °C).

The stress-strain curves and therefore the crushing strength of EG particles are shown in Figure 4. It is apparent that the preheating time of EG particles is an important parameter. Particles preheated for 6 min are referred to as stable (StEG); because, their properties (i.e., $\rho_{B}=$ 0.20 g/cm^3^, $\sigma_{\mathrm{PL}}=$ 1.5 MPa) do not significantly change compared to as-received particles (i.e., $\rho_{B}=$ 0.19 g/cm^3^, $\sigma_{\mathrm{PL}}=1.4$ MPa). For the preheating time of 20 min, the average plateau stress increases to 2.5 MPa and the bulk density to 0.27 g/cm^3^. 

### 2.2. Structural Characteristics of MSF

The following section addresses the structural characteristics of the manufactured MSF. Of particular interest is the possible existence of voids between particles due to incomplete infiltration. Voids introduce localised weaknesses into the foam and may act as seeds for macroscopic failure. Table 1 shows the bulk, particle, and true density of stable and shrunk EG particles (referred to StEG and ShEG respectively). Using this data in conjunction with Equations (2)–(5), the volume fraction of matrix $\left(F_{M}\right)$, particles $\left(F_{P}\right)$, and voids $\left(F_{V}\right)$ can be calculated. The results are listed in Table 2. 

It is apparent that voids exist in all foam samples. The shrinkage of EG particles decreases the void volume fraction from an average of $F_{V}=$ 8.8% for stable particles to 3.6% for shrunk particles. 

**Figure 5:**
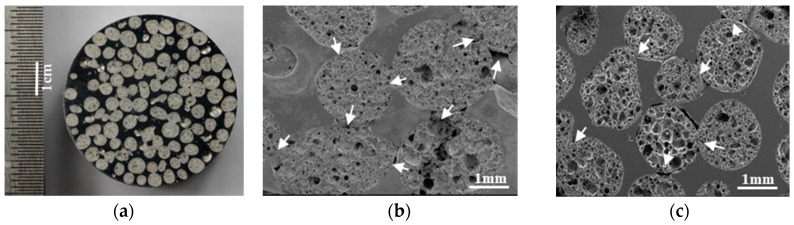
(**a**) EG-MSF cross-section; (**b**) SEM Images of StEG-MSF; and (**c**) SEM image of ShEG-MSF.

Figure 5a shows the cross-section of an EG foam illustrating a good distribution of particles through the foam. Additionally, it is obvious that all particles remained intact during infiltration. There is no sign of particle breakage or unintended infiltration of EG particles by the Al melt. Figure 5b,c show SEM cross-sections of EG-MSF with stable and shrunk EG particles, respectively. Stable particles do not undergo significant deformation and mostly retain their spherical shape. As a result, they exhibit small contact areas neighboured by narrow channels. It can be seen in Figure 5b that the aluminium melt fails to infiltrate some of these narrow channels resulting in inter-particle voids. In contrast, shrunk EG particles flatten at their contact area and the narrow channels mostly disappear. This likely results in a decreased void volume fraction, which supports the findings from Table 2. Furthermore, despite shrunk EG becoming smaller (see Figure 5c), slightly higher values of the EG volume fraction $F_{P}$ are observed for shrunk EG foam samples. A likely explanation is the denser packing of shrunk EG due to localized flattening at contact points. The matrix volume fraction of shrunk particles is also increased due to improved infiltration. 

Next, the possible reaction between the EG particles and the Al matrix was investigated. EG is largely composed of SiO_2_ which can react with aluminium through the following reaction [15]:

(1) $${4\mathrm{Al}(l)\;+\;3\mathrm{SiO}}_{2}{(s)\;=\;2\mathrm{Al}}_{2}O_{3}(s)\;+\;3\mathrm{Si}(s)\;\Delta {G\;=\;-310\;\mathrm{to}\;-330\;\mathrm{kJ}\cdot \mathrm{mol}}^{-1},\;700–850\;{}^{\circ}C$$

**Figure 6:**
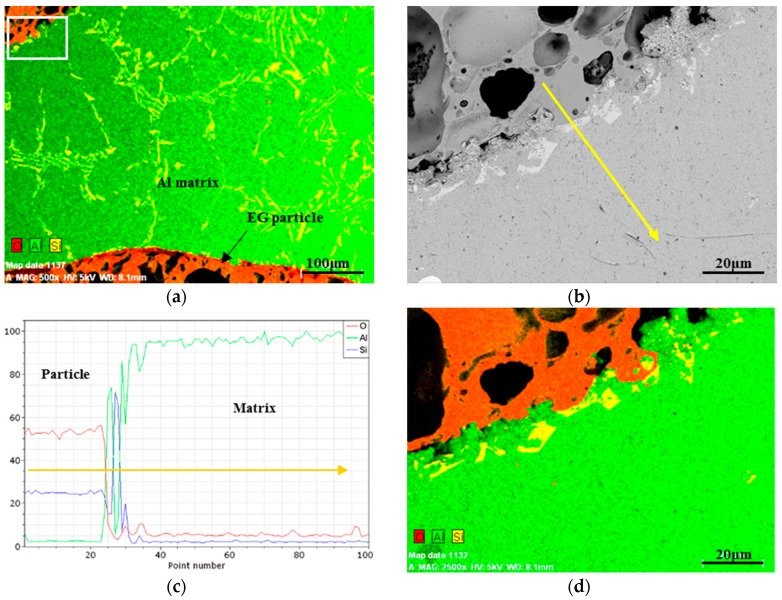
(**a**) BSE image of base metal; (**b**) BSE image of the interface; (**c**) EDS line-scan profiles of the EG-A356 syntactic foam; and (**d**) elemental analysis image X-ray map of the interface area.

The A356 aluminium has a eutectic microstructure comprised of an aluminium-rich phase and silicon grown between the primary dendritic networks. Figure 6a shows a backscattered electron (BSE) image of the A356 matrix between two EG particles. The eutectic phases are homogeneously distributed through the whole metal matrix. The bright areas show the Si network in the A356 aluminium microstructure. Chemical analysis was performed on the area marked with a white rectangle in Figure 6a. The occurrence of chemical reaction between the aluminium matrix and the filler particles is more significant at higher infiltration temperatures and exposure times [31,32]. Accordingly, EDS elemental analysis of the interfacial zone was performed on a sample with shrunk particles which were exposed to the melt for a longer time. Figure 6b shows a backscattered electron image of the investigated interfacial area of the polished sample. Figure 6c shows the measured elemental concentration of oxygen (O), aluminium (Al), and silicon (Si) along the yellow arrow. The high concentrations Si and O remain constant within the EG particle. The concentration of oxygen drops dramatically across the interface while the aluminium concentration increases towards the matrix material. The yellow path crosses over a eutectic Si grain and causes inverse fluctuations of Al and Si. 

In the case of chemical reaction between EG particles and the Al matrix, an ${\mathrm{Al}}_{2}O_{3}$ layer grows into the Al matrix and a high concentration of oxygen would be detectable in the areas adjacent to the interface. However, no change in O concentration was found within the metal matrix as shown in Figure 6c,d. Accordingly, it can be concluded that there is no significant chemical reaction in the current system.

### 2.3. Compressive Properties of EG-MSF

**Figure 7:**
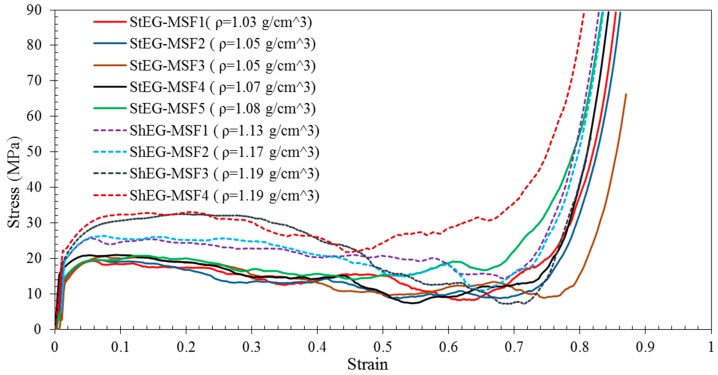
Compressive stress-strain curves for stable (solid lines) and shrunk (dashed lines) EG-MSF.

Five samples of stable EG-MSF (StEG-MSF) and four samples of shrunk EG-MSF (ShEG-MSF) were tested on a Shimadzu testing machine. The results are shown in Figure 7. The stress-strain curves exhibit the typical behaviour of metallic syntactic foams comprising an elastic deformation regime, plateau region, and densification. Following elastic deformation, plasticity commences smoothly with no stress drop after yield for both stable and shrunk EG MSF. Then, the stress remains relatively constant during deformation until it increases sharply due to densification of the foam. Comparing foams with shrunk and stable EG-MSF it becomes apparent that particle shrinkage (dashed lines) distinctly increases strength whilst densification is only slightly affected. All foams show large plateau regions and densification strains higher than 70%. Consequently, this foam is expected to exhibit high performance in energy-absorbing applications. 

**Figure 8:**
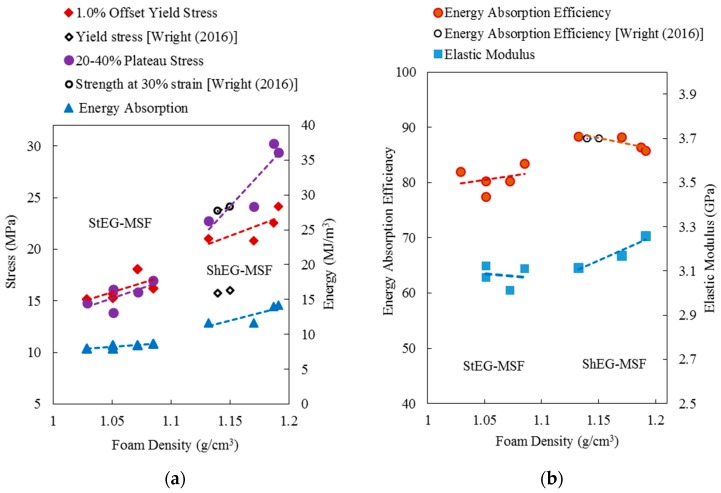
Mechanical properties of EG-MSF versus with the foam density: (**a**) plateau stress and energy absorption; and (**b**) elastic modulus and energy absorption efficiency. Additional data points received from Wright et al. [28].

In Figure 8, the 1.0% offset yield stress, plateau stress, and energy absorption (that calculated using Equation (6)) are plotted versus foam density. As expected, all material properties increase with density. The well-known explanation is that higher metal volume fractions result in both increased density and load bearing capacity of foams [30]. For lightweight applications, the specific material properties (i.e., material properties normalized by material density) are of particular interest. The average specific yield stress of ShEG-MSF (18.9 kJ/kg) is 21% higher than stable EG-MSF (14.9 kJ/kg). This deviation increases to 36% for the specific plateau stress, i.e., 14.7 MPa/kg (stable) versus 22.8 MPa/kg (shrunk). Finally, the average specific energy absorption of ShEG-MSF is 28% higher compared with StEG-MSF. It can be concluded that the shrinkage of EG particles increases strength and energy absorption capacity of foams per unit mass and, hence, is attractive for applications where minimum mass is required. Likely explanations are (i) the increased crushing strength of shrunk EG particles (see Figure 4); and (ii) a lower void volume fraction (see Table 2). Voids, i.e., the absence of the load bearing metallic matrix, cause local weaknesses within the foam structure. Adjacent particles and matrix must bear additional load and are likely to fail, thus creating a new defect within the structure. As a result, failure bands may originate from such voids and propagate through large sections of the foam sample at relatively low external loads thus decreasing macroscopic foam strength (see Section 2.4). 

Figure 8b shows the elastic unloading modulus and energy absorption efficiency (that calculated using Equation (7)) plotted versus foam density. Interestingly, both material properties of StEG-MSF show little variation and exhibit approximately constant values within the considered density range. In comparison, the elastic unloading modulus of ShEG-MSF increases systematically (from 3.1 GPa to 3.25 GPa) with foam density. Conversely, a small decline in energy absorption efficiency (from 88 to 85%) is found for the higher density ShEG-MSF. The decreased energy absorption efficiency is caused by the higher oscillations of the plateau stress clearly visible in Figure 7. On average, ShEG-MSF exhibit slightly higher elastic unloading moduli and energy absorption efficiencies compared with StEG-MSF.

It is of interest to compare the mechanical properties of the tested samples with EG MSF syntactic foams made by Wright et al. [28] (AlSi12/EG). They manufactured syntactic foams using preheating temperatures up to 600 °C. The resulting density 1.12–1.13 g/cm^3^ coincided with the ShEG MSF of the current study. Their yield stress (empty diamond markers) is distinctly below the compressive yield stress of ShEG-MSF. However, the stresses at 30% strain (no plateau stress was provided but this value is considered to be a close equivalent) and energy absorption efficiencies of Wright’s foams are similar to the plateau stress and energy absorption efficiency of ShEG-MSF. 

### 2.4. Deformation Mechanism of EG-MSF

All samples manufactured under the same conditions revealed similar deformation behaviour under quasi-static compression (see Figure 7). However, a wider scatter of stress and material properties was found in the case of shrunk EG MSF. 

**Figure 9:**
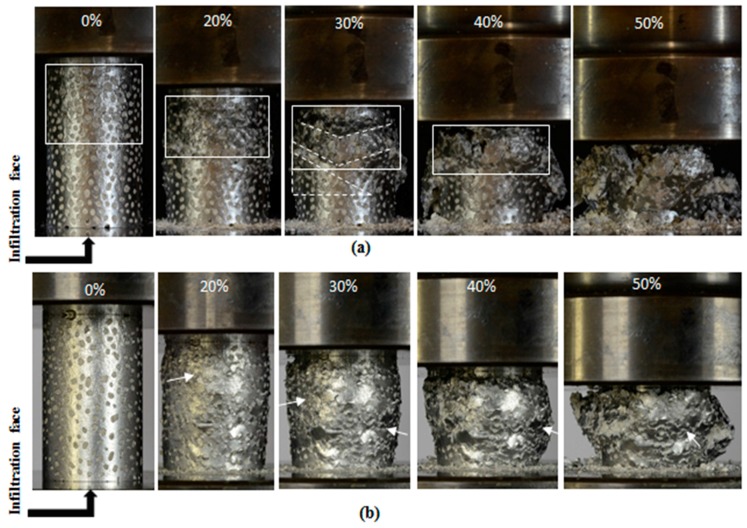
Compressive deformation mechanism of EG/A356 syntactic foam: (**a**) StEG-MSF4; and (**b**) ShEG-MSF3.

Figure 9a,b show optical images of stable and ShEG-MSF samples during compression testing. Comparing the undeformed foams, one can see that the concentration of uniformly-dispersed EG particles is higher on the surface of stable EG foam. The cell windows to the surface of ShEG-MSF are smaller both in number and size. One likely explanation is that the shrinkage of the particles results in a gap between the surface particles and the mould wall. This gap is filled with molten metal during infiltration and turns to a solid shell with a small number of cell windows around the sample. 

StEG-MSF showed different deformation behaviour than ShEG foams. In Figure 9a localized deformation resulted in the early formation of shear bands at 20% macroscopic strain. These shear bands are V shaped at 30–45° [33] to the horizontal direction as marked in the figure with white lines. A dead zone with no obvious deformation was detected underneath these bands (shown by a white triangle). Deformation was concentrated in the vicinity of the multiple shear bands. At the same time, the rest of the sample remained largely undeformed. On the other hand, ShEG-MSF showed relatively uniform deformation. Figure 9b shows that shrunk EG-MSF samples deformed in a layer by layer collapse mechanism (indicated by white arrows) without the catastrophic formation of macroscopic shear bands; and, thus, no dead zone was observed. Some minor shear bands formed during the final stages of deformation (50%) and caused disintegration of the sample during densification. 

## 3. Methodology 

### 3.1. Characterisation of EG Particles

Expanded glass (EG) particles were supplied by Dennert Poraver GmbH. Mozartweg 1, 96132 Schlüsselfed, Germany [23]. A sieving process was used to separate particles with a minimum width equal to the mesh sizes of 2–2.8 mm. The bulk density $\left(\rho_{B}\right)$ of EG particles was obtained by measuring the mass and volume of packed particle beds after tapping the container 300 times. The bulk density was calculated by dividing the macroscopic volume ($V_{P})$ by the combined particle mass ($m_{P})$, i.e., $\rho_{B}=V_{P}/m_{P}$. The particle density ($\rho_{P})$ was obtained using the fine powder technique. To this end, fine flour was poured into a graduated cylinder (27 mm in diameter), tapped 300 times, and its volume was recorded. Next, 6 g of EG particles was mixed with the same flour batch and poured into the graduated glass cylinder. The volume of the mixture was re-measured after 300 taps. The combined particle volume was calculated as the volume difference ΔV prior to and after EG particles addition. Particle density was then obtained using $\rho_{P}=\Delta V/m_{P}$. The true density $\rho_{T}$ (the density of solid part of particle) was determined based on the chemical composition of the glass.

EG particles exhibit a relatively low glass transition temperature of 570–600 °C because of the low alkali oxide content which limits cross-networking in the glass [34]. Heating of particles to their transition temperature triggers particle shrinkage. To study the shrinkage phenomenon of EG at different temperatures, a graphite crucible was filled with EG particles and was placed in a preheated furnace. The packed EG beds were exposed to furnace temperatures between 600 °C and 800 °C with 25 degree increments. The crucible was removed from the furnace after 20 min and the (linear) shrinkage was calculated as $S=\left(h_{O}-h_{F}\right)/h_{O}$ where $h_{O}$ and $h_{F}$ stand for initial and final height of the EG column, respectively. A similar test was conducted for EG particles at a constant furnace temperature of 700 °C (i.e., the selected casting temperature of Al356) and the exposure time was varied. In these tests, a thermocouple was inserted into the packed particle bed to measure the local temperature, i.e., particle temperature.

The crushing strength of EG particles was examined by confined compression testing. To this end, a steel cylinder (ø30 mm inside) was filled with EG particles to a height of 40 mm and tapped to ensure dense particle packing. Compression tests were conducted for as-received, stable (heated for 6 min at 700 °C) and shrunk (heated for 20 min at 700 °C) EG particles. Three compression tests were conducted for each particle type by pressing a steel plunger into the particle bed until its initial height was decreased by 70%. Tests were conducted on a Shimadzu testing machine with 5 kN load cell at a constant axial cross-head speed of 0.5 mm/min.

### 3.2. EG-MSF Sample Preparation

A356 aluminium alloy was used as the matrix material for manufacture the metal syntactic foams. According to the supplier material datasheet (Hayes Metals Pty Ltd., Riverstone NSW 2765, Australia), the chemical composition comprises wt% 92.16 Al, 7.2 wt% Si, 0.4 wt% Mg, 0.1 wt% Fe, and 0.12 wt% Ti. Following the mixing rule, the calculated true density of this aluminium alloy is $\rho_{A356}=$ 2.68 g/cm^3^. Thanks to its short solidification time, low shrinkage, hot cracking resistance, and excellent castability, A356 alloy is the most widely used casting alloy in Al foundries. These properties are mainly due to its high silicon content [35]. The presence of Mg and Si improves the mechanical properties in both as received and heat-treated conditions [36]. Furthermore, the presence of Mg improves the EG wettability of molten A356 [37].

The expanded glass—metal matrix syntactic foam (EG-MSF) samples were produced using the counter-gravity pressure infiltration technique described previously [15]. EG particles were filled into a graphite mould in five equally-sized batches and tapped eight times after each filling step to obtain a densely packed EG particle bed. One stainless steel mesh was positioned at the bottom of the mould to avoid blockage of the ventilation hole and another one at the top to prevent the displacement of EG particles during melt infiltration. This assembly was preheated in a muffle furnace to 700 °C and held for either 6 min or 20 min. Simultaneously, A356 alloy was melted in a graphite crucible and heated to 700 °C. The EG particle assembly was inverted and pushed into the metallic melt to infiltrate the voids between the particles. Following forced-air cooling, the cylindrical samples were removed from the mould and their upper and lower surfaces were machined to remove the stainless steel meshes. The final height of the samples varied between 36 mm and 40 mm.

### 3.3. EG-MSF Characterisation

The microstructure of EG-MSF samples was examined using scanning electron microscopy (SEI-Philips XL30 SEM, Eindhoven, Netherlands). EG-MSF samples were first polished with SiC paper, followed by 6 μm and 1 μm water-based diamond suspensions. The samples were then placed in an ultrasonic bath for 20 min and dried in an oven for 24 h. Chemical analysis of the samples was obtained by energy-dispersive X-ray spectroscopy (EDS) within the SEM. 

The metal syntactic foam density $\left(\rho_{\mathrm{MSF}}\right)$ was calculated by the division of foam mass by its volume, i.e.:

(2) $$\rho_{\mathrm{MSF}}=\frac{m_{\mathrm{MSF}}}{V_{\mathrm{MSF}}}$$

The combined particle mass $\left(m_{P}\right)$ of the EG particles inside a MSF sample can be estimated using the syntactic foam volume and the particle bulk density $\left(\rho_{B}\right)$:

(3) $$m_{P}=\rho_{B}\times V_{\mathrm{MSF}}$$

Next, the volume fraction of matrix $\left(F_{M}\right)$, particles $\left(F_{P}\right)$, and voids $\left(F_{V}\right)$ can be calculated using:

(4) FM=(mMSF−mP)ρA356VMSF

(5) FP=mPρPVMSF

(6) $$F_{V}=1-F_{M}-F_{P}$$

where $\left(\rho_{P}\right)$ is the particle density that was obtained using the fine powder technique. Voids here refers to the inter-particles volume that is not infiltrated with molten metal. Based on the chemical composition of EG particles [15], their true solid density $\left(\rho_{T}\right)$ is calculated as 2.81 g/cm^3^. 

To determine the mechanical properties of EG-MSF samples, quasi-static compression tests were conducted following the ISO 13314 standard [38]. A computer-controlled uni-axial Shimadzu testing machine with a 50 kN capacity load cell compressed samples at a constant crosshead speed of 1 mm/min. Lubrication was applied to the parallel compression platens in order to minimize friction between the sample faces and loading platens. A controlled unloading cycle was used to measure the elastic unloading modulus. To this end, a low density sample was tested first and its plateau stress $\left(\sigma_{\mathrm{PL}}\right)$ was measured as the arithmetic mean of stresses between 20% and 40% of macroscopic strain. The rest of the samples were unloaded once 70% of the given plateau stress was reached. The unloading continued to $0.2\times \sigma_{\mathrm{Pl}}$. Following partial unloading, the sample was further compressed until the machine load limit (50 kN) was reached. The measured unloading slope is defined as the elastic unloading modulus. The unloading modulus is considered completely elastic and not affected by localized plasticity and settling effects; therefore, it provides a good approximation of the rigidity of the foam. The 1% offset yield stress was determined to estimate the onset of plastic deformation. The volumetric energy absorption capacity (W) of EG-MSF can be calculated by integrating the stress-strain data up to 50% of compressive strain (e) according to:

(7) $$W=\;\int_{0}^{e_{50\%}}\sigma de$$

where σ and e are the compressive stress and strain, respectively. Another important material parameter is the energy absorption efficiency (η). It is defined as the ratio of actual and ideal energy absorption. An ideal absorber immediately reaches its plateau stress, which remains constant up to densification. Thus, η is defined as:

(8) $$\eta =\;\frac{W}{\sigma_{\mathrm{MAX}}e_{50\%}}$$

where $\sigma_{\mathrm{MAX}}$ is the foam’s maximum stress up to 50% strain.

## 4. Conclusions 

The present work addressed the structural and mechanical characterisation of expanded-glass metal syntactic foam (EG-MSF). EG particle shrinkage was utilized as a design parameter to modify the mechanical properties of the resulting syntactic foam. It was found that particle shrinkage is widely prevented by limiting the exposure time at high furnace temperatures (>600 °C). However, controlled particle shrinkage allowed the increase of all material properties considered, most importantly the 1% offset yield stress (28.8% increase) and plateau stress (41.8% increase). Lightweight applications require high specific material properties and a similar trend was observed, i.e., a 21.2% increase of the specific 1% offset yield stress and a 35.5% increase of the specific plateau stress for shrunk EG-MSF. This demonstrates that the foam density increase caused by particle shrinkage is outweighed by the improvement of mechanical performance. Likely explanations for the significantly improved material strength are the increased shrunk particle crushing strength and a decreased void volume fraction. It can be concluded that EG particle shrinkage is an efficient strategy to improve the mechanical performance of EG-MSF. 

Future research could address the effect of EG particle size and its chemical composition on the syntactic foam properties. Furthermore, the usage of different metallic matrix materials and heat treatment can be probed to tailor the mechanical properties of the material to specific engineering applications. 

## Figures and Tables

**Table 1:** EG particles densities.

Particles Group	Bulk Density $\left(\rho_{B}\right)\;$ g/cm^3^	Particle Density $\left(\rho_{P}\right)$ g/cm^3^	Packing Density ($\rho_{B}/\rho_{P})$	True Density $\left(\rho_{T}\right)$ g/cm^3^
As-received EG	0.19	0.33	0.58	2.81
Stable EG	0.20	0.34	0.59	2.81
Shrunk EG	0.27	0.43	0.63	2.81

**Table 2:** Physical properties of EG-MSF samples (St and Sh mean stable and shrunk EG, respectively).

Sample	Foam Weight $m_{\mathrm{MSF}}$ (g)	Foam Volume $V_{\mathrm{MSF}}$ (cm^3^)	Foam Density $\rho_{\mathrm{MSF}}$ (g/cm^3^)	Matrix Volume Fraction $F_{M}$ (%)	EG Volume Fraction $F_{P}$ (%)	Void Volume Fraction $F_{V}\;$ (%)
StEG-MSF1	22.30	21.65	1.03	30.97	60.61	8.42
StEG-MSF2	23.86	22.69	1.05	31.77	58.82	9.41
StEG-MSF3	23.98	22.79	1.05	31.80	58.82	9.38
StEG-MSF4	24.33	22.68	1.07	32.57	58.82	8.61
StEG-MSF5	24.65	22.68	1.08	33.09	58.82	8.09
ShEG-MSF1	22.48	19.85	1.13	32.17	62.79	5.04
ShEG-MSF2	24.71	22.11	1.17	33.60	62.79	3.61
ShEG-MSF3	25.06	21.11	1.19	34.22	62.79	2.99
ShEG-MSF4	23.40	21.10	1.19	34.38	62.79	2.83
